# Meta-Analysis of Multiple Sclerosis Microarray Data Reveals Dysregulation in RNA Splicing Regulatory Genes

**DOI:** 10.3390/ijms161023463

**Published:** 2015-09-30

**Authors:** Elvezia Maria Paraboschi, Giulia Cardamone, Valeria Rimoldi, Donato Gemmati, Marta Spreafico, Stefano Duga, Giulia Soldà, Rosanna Asselta

**Affiliations:** 1 Dipartimento di Biotecnologie Mediche e Medicina Traslazionale, Università degli Studi di Milano, Via Viotti 3/5, Milan 20133, Italy; E-Mails: elvezia.paraboschi@unimi.it (E.M.P.); giulia.cardamone@studenti.unimi.it (G.C.); 2 Department of Biomedical Sciences, Humanitas University, Via Manzoni 113, Rozzano, Milan 20089, Italy; E-Mails: valeria.rimoldi@humanitasresearch.it (V.R.); stefano.duga@hunimed.eu (S.D.); rosanna.asselta@hunimed.eu (R.A.); 3 Humanitas Clinical and Research Center, Via Manzoni 56, Rozzano, Milan 20089, Italy; 4 Center Haemostasis & Thrombosis, Department of Medical Sciences, Corso Giovecca 203, University of Ferrara, Ferrara 44121, Italy; E-Mail: d.gemmati@unife.it; 5 Department of Transfusion Medicine and Hematology, Azienda Ospedaliera della Provincia di Lecco, Alessandro Manzoni Hospital, Via dell’Eremo 9/11, Lecco 23900, Italy; E-Mail: ma.spreafico@ospedale.lecco.it

**Keywords:** alternative splicing, microarray, peripheral blood mononuclear cells, autoimmune neurodegenerative disorder, multiple sclerosis

## Abstract

Abnormalities in RNA metabolism and alternative splicing (AS) are emerging as important players in complex disease phenotypes. In particular, accumulating evidence suggests the existence of pathogenic links between multiple sclerosis (MS) and altered AS, including functional studies showing that an imbalance in alternatively-spliced isoforms may contribute to disease etiology. Here, we tested whether the altered expression of AS-related genes represents a MS-specific signature. A comprehensive comparative analysis of gene expression profiles of publicly-available microarray datasets (190 MS cases, 182 controls), followed by gene-ontology enrichment analysis, highlighted a significant enrichment for differentially-expressed genes involved in RNA metabolism/AS. In detail, a total of 17 genes were found to be differentially expressed in MS in multiple datasets, with *CELF1* being dysregulated in five out of seven studies. We confirmed *CELF1* downregulation in MS (*p* = 0.0015) by real-time RT-PCRs on RNA extracted from blood cells of 30 cases and 30 controls. As a proof of concept, we experimentally verified the unbalance in alternatively-spliced isoforms in MS of the *NFAT5* gene, a putative *CELF1* target. In conclusion, for the first time we provide evidence of a consistent dysregulation of splicing-related genes in MS and we discuss its possible implications in modulating specific AS events in MS susceptibility genes.

## 1. Introduction

The role of RNA metabolism in controlling fundamental functions in the central nervous system (CNS) and in the pathogenesis of neurodegenerative disorders is being increasingly appreciated [1]. The discovery of mutations in key RNA binding proteins in several human neuronal-based diseases has firmly placed RNA processing/metabolism as central to disease etiology [2]. Alternative splicing (AS) is a post-transcriptional mechanism that expands the RNA information content through the expression of multiple different transcripts from individual genes. It is estimated that the vast majority (up to 97%) of protein coding genes express multiple mRNAs through AS [3,4]: ~80% of the variability generated by AS involves the coding regions, whereas the remaining 20% might affect cis-acting elements that control mRNA stability, translation efficiency, and localization. Moreover, two thirds of AS events are predicted to introduce premature termination codons, which trigger mRNA degradation by the nonsense-mediated mRNA decay (NMD) pathway [5]. Considering the complexity and the finely-tuned regulation of AS, it is not surprising that splicing alterations can directly cause diseases, modify the severity of disease phenotypes, or be linked to susceptibility to complex disorders. The mechanisms causing altered splicing can involve the disruption of cis-acting elements within the affected gene, as well as the dysregulation of trans-acting splicing factors. The distinction between cis- and trans-acting effects has important mechanistic implications: cis elements have a direct impact on the expression of only one gene, whereas trans-acting factors affect the expression of multiple genes [3].

Multiple sclerosis (MS; OMIM#126200) is a clinically heterogeneous, inflammatory, demyelinating disease of the CNS, with an autoimmune pathogenesis and complex inheritance [6]. Despite longstanding and intensive efforts, genetic and environmental risk factors still remain largely unknown [7,8,9]. Few studies have investigated the link between AS dysregulation, aberrant splice-regulatory protein expression, and MS. The first study monitoring differential expression and AS patterns in whole blood revealed no substantial changes in full-length transcript levels between MS patients and controls, but a highly significant differential expression of a large number of exons, with at least 340 genes predicted to undergo AS. Indeed, while unsupervised hierarchical clustering applied to full-length transcripts could not distinguish MS cases from controls, clustering of alternatively-spliced exons well discriminated between the two groups. This suggested that AS patterns could be used as biomarkers, particularly during quiescent times of the disease [10]. Besides these preliminary data, several pieces of evidence point to a potential role of AS in MS [11]. Genetic susceptibility to MS has been mapped to many SNPs, but the description of the underlying functional mechanism is often missing. However, when the functional variant has been characterized, it invariantly affected the gene splicing process [11,12]. Even the strongest risk factor for MS (*i.e.*, the HLA DRB1*1501-DQB1*0602 haplotype [7,8,9] seems to exert its predisposing role by determining an allele-specific splicing pattern at the *DQB1 locus* [13].

The potential role of AS in MS pathogenesis is also strengthened by the observation that AS can modulate immunogenicity by: (i) creating novel antigenic epitopes; (ii) altering antigen surface accessibility; and (iii) enabling the expression of autoantigens; and (iv) altering patterns of post-translational modifications [11,14]. Thus, AS may be responsible for the generation of untolerized epitopes, and, as a result, of autoimmunity.

Finally, the potent immunogenicity of heterogeneous nuclear ribonucleoproteins (hnRNPs), which act as activators and/or repressors of splicing, was demonstrated to play a pathogenic role in autoimmune disorders [15,16] and neurodegenerative diseases, including MS, where anti-hnRNP-A1/A2/B1 antibodies have been identified [17,18]. Importantly, the autoimmune reaction against hnRNPs might contribute to neurodegeneration: transfection of anti-hnRNP-A1 antibodies into neurons resulted in both severe neuronal injury and in changes in the levels of transcripts potentially regulated by hnRNP-A1 [19].

With these premises, we aimed at exploring a possible generalized alteration of AS control in relapsing-remitting (RR) MS (the most common form, representing ~80% of all diagnosed patients) by determining whether the disease status is associated with changes in splice-regulatory protein gene expression. In doing this, we applied a combined approach based on *in-silico* analyses of microarray datasets, publicly available through the Gene Expression Omnibus (GEO) repository, as well as on *in-vivo* measurements on RNA extracted from peripheral blood mononuclear cells (PBMCs) of RR-MS cases and controls.

## 2. Results and Discussion

### 2.1. Dataset Selection

We selected seven microarray datasets from MS case/control cohorts publicly available at the NCBI GEO database (Table 1). In all cases, gene expression experiments were performed on RNA extracted from blood-derived samples (either PBMCs, whole blood, peripheral blood T-cells, or naïve CD4^+^ T cells). These datasets account for a total of 372 subjects (190 RR-MS patients and 182 controls). Three studies used the Affymetrix Human Genome U133 Plus 2.0 microarray platform, three were conducted with the Affymetrix Human Exon 1.0 ST Array, and one was based on the Illumina HumanHT-12 V3.0 chip.

### 2.2. The “Nuclear mRNA Splicing” Pathway Is Consistently Dysregulated in MS

To search for pathways specifically dysregulated in MS, we performed a gene ontology (GO) enrichment analysis with DAVID (Database for Annotation, Visualization and Integrated Discovery) using, for each dataset, the top 3000 up/downregulated genes, as retrieved from GEO2R. The main results are summarized in Table 2, Tables S1 and S2. We found a total of 141 significantly-enriched clusters (about 20 for each dataset). Two pathways consistently emerged from the majority of datasets: (i) the group of “Nuclear mRNA splicing” functionally-related genes (five out of seven datasets; two groups surviving the correction for multiple testing); and (ii) the “NADH dehydrogenase complex” group (four out of seven datasets; two groups surviving the correction for multiple testing). Interestingly, in two out of seven datasets we also observed the recurrence of another GO functional category related to mRNA metabolism, *i.e.*, the “RNA localization” cluster, reinforcing the concept that altered mRNA processing could be a specific signature of MS (Table S2H).

**Table 1:** Characteristics of multiple sclerosis (MS)-related datasets included in the study.

Dataset (accession number)	Array Type	MS Cases/Controls	Origin	% Females (Cases/Controls)	Age (Years)	Notes on MS Patients Included	Tissue	Ref.
GSE21942	Affymetrix Human Genome U133 Plus 2.0 Array	10/15	Finland	100/100	Mean age cases: 54.2 Mean age controls: 71.6	4 patients under treatment	Peripheral blood mononuclear cells	[20]
GSE41848	Affymetrix Human Exon 1.0 ST Array	54/38 (discovery dataset)	n.a.	71/74	Median age cases: 43 (range: 22–66) Median age controls: 46 (range 26–66)	Only RR-MS untreated cases included in the analyses	Whole blood	[21]
GSE41849	Affymetrix Human Exon 1.0 ST Array	21/22 (replication dataset)		66/59	Median age cases: 45 (range 23–61) Median age controls: 42 (range 27–61)			
GSE41890	Affymetrix Human Gene 1.0 ST Array	22/24	n.a.	56/52	Mean age cases: 40 (range 23–66) Mean age controls: 38 (range 23–57)	RR-MS patients in remitting phase	Peripheral blood leukocytes	[22]
GSE17048	Illumina HumanHT-12 V3.0 Expression BeadChip	36/45	Australia	80/64	Mean age cases: 48.5 (range 29–65) Mean age controls: 48.5 (range 23–77)	Only RR-MS untreated cases included in the analyses	Whole blood	[23,24]
GSE43592	Affymetrix Human Genome U133 Plus 2.0 Array	10/10	Sweden	40/40	Mean age cases: 38 Mean age controls: 40	RR-MS	Peripheral blood T-cells	[25]
GSE13732	Affymetrix Human Genome U133 Plus 2.0 Array	37 */28	92% Caucasian	74/64	Mean age cases: 37 Mean age controls: 35	-	Naïve CD4^+^ T cells	[26]

* Clinically isolated syndrome (CIS), the earliest clinical manifestation of MS. Abbreviations: n.a., not available; RR, relapsing remitting MS.

As a control, the same analysis was performed on a similar group of datasets (seven studies, 390 cases and 348 controls, blood-derived RNA samples) involving a disease with a completely different etiology, *i.e.*, coronary artery disease and its major complication, myocardial infarction. This analysis did not show a pattern of enriched functionally-related genes similar to that observed for MS (Table S3).

Finally, to confirm the observed pathway enrichment, we performed an alternative analysis based on a different approach: in this case, we first conducted a meta-analysis by using the INMEX (Integrative Meta-analysis of Expression Data) software, and the identified consistently differentially-expressed genes were subsequently subjected to a GO enrichment analysis. We found 107 significantly enriched GO clusters (*p* < 0.0005), with the “RNA binding” category being among the top five (*p* = 7.7 × 10^−23^) (Table S4), once again underlying the possible role of RNA processing in the pathogenesis of MS.

**Table 2:** Summary of the DAVID results.

Dataset (Accession Number)	No. of Identified Cluster *	No. of Significantly Enriched Clusters **	Enrichment Score Range (max–min) ***	“Nuclear mRNA Splicing” Category		“NADH Dehydrogenase Complex” Category	
				Cluster Rank	Enrichment Score	Cluster Rank	Enrichment Score
GSE21942	218	27	12–1.34	1	12 ****	8	3 ****
GSE41848	246	37	4.2–1.32	7	2.67	6	2.97
GSE41849	214	9	5.52–1.35	-	-	1 and 2	5.52 and 5.1 ****
GSE41890	185	10	5.1–1.47	-	-	-	-
GSE17048	174	10	2.41–1.34	4	2.16	10	1.34
GSE43592	222	23	5.24–1.32	10	1.82	-	-
GSE13732	199	25	9.06–1.33	3	4.34 ****	-	-

* Total number of groups of functionally-related genes identified by DAVID through the integration of data from dozens of databases (or details see [27]); listed numbers include both significantly and not-significantly enriched groups; ** Clusters significantly enriched are those showing a DAVID enrichment score >1.3 (corresponding to *p* < 0.05 calculated through a modified Fisher exact test, one tailed [27]); *** It is provided the score range for the significantly enriched groups; and **** Cluster surviving the correction for multiple testing (Benjamini correction). DAVID, Database for Annotation, Visualization and Integrated Discovery (http://david.abcc.ncifcrf.gov/).

### 2.3. The CELF1 Gene Emerges as a Major Splicing-Regulator Altered in MS

The “Nuclear mRNA splicing” GO category comprises a total of 126 genes (source: the KEGG catalogue). To identify shared dysregulated transcripts belonging to this category among the five datasets, we intersected the corresponding gene lists, which were composed of 54, 39, 26, 30, and 39 genes, respectively (Table S2). We found one gene present in all five clusters (*CELF1/CUGBP1*, CUG triplet repeat, RNA binding protein 1), one present in four out of five clusters (*MBNL1*, muscleblind-like splicing regulator 1), and 15 genes present in three out of five clusters (Table 3). A meta-analysis, performed with the INMEX tool, allowed us to verify the direction (up/downregulation) of the observed altered expression of the most interesting genes (*i.e.*, those shared by at least three datasets) (Table 3).

**Figure 1:**
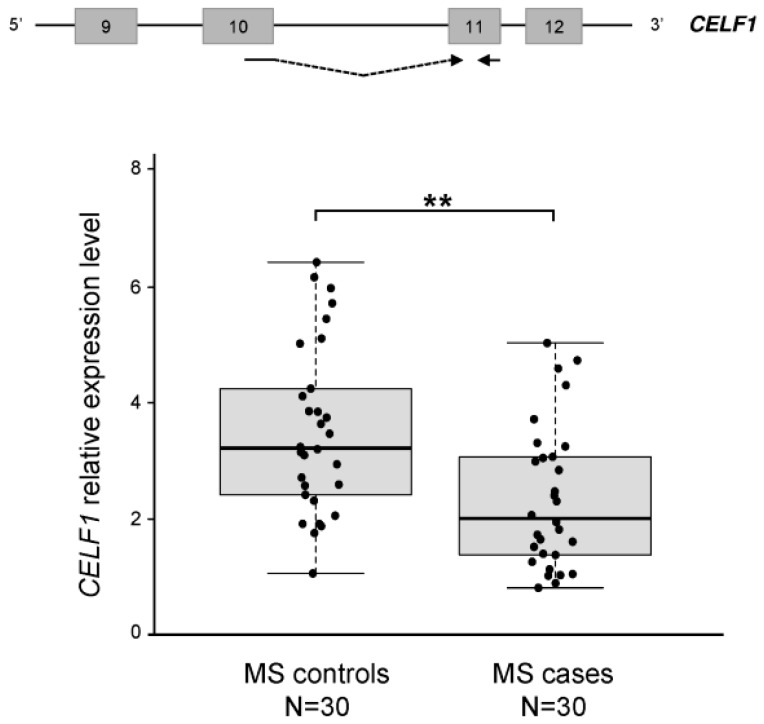
CUGBP Elav-like family member 1 (*CELF1*) expression levels in peripheral blood mononuclear cells (PBMCs) of MS patients and controls. Expression levels were measured by semi-quantitative real-time RT-PCRs in 30 MS patients and 30 controls, using primers located in exons 10 and 11, as depicted in the scheme above boxplots. Boxplots show *CELF1* expression levels according to disease status. Boxes define the interquartile range; the thick line refers to the median. Results are presented as normalized rescaled values. Significance level for differences between groups was calculated by a *t*-test. **: *p* < 0.005.

To confirm—at least in part—these *in-silico* results, we measured by semi-quantitative real-time RT-PCR assays the expression level of *CELF1*, the only transcript dysregulated in five out of five datasets. Real-time experiments were performed on RNA extracted from PBMCs of 30 RR-MS patients (free from treatments) and 30 healthy controls. *CELF1* was significantly overexpressed (on average 1.5-fold) in healthy individuals respect to RR-MS patients (*p* = 0.0015) (Figure 1), confirming the upregulation observed in controls in the microarray data (Table 3).

### 2.4. Effect of Splicing Alteration on Genotype-Dependent AS Events

Due to the observed generalized dysregulation of genes directly involved in splicing control, we expect that this could potentially reflect on the AS of a number of genes, altering their pattern of alternative isoforms. The presence of a polymorphism located directly at splice sites or in *cis*-acting splicing regulatory elements (*i.e.*, possibly modifying the splicing pattern *per se*) could in theory exacerbate and make detectable the effect of the splicing dysregulation, with the polymorphism acting as genotype-dependent “AS sensor”.

**Table 3:** The 17 splice-regulatory genes found dysregulated in at least three MS datasets enriched for the gene-ontology category “nuclear mRNA splicing”.

Gene Symbol	Gene Name	No. of Datasets in Which the Gene Is DE	Combined T Stat *	Combined *p* Value
CELF1/CUGBP1	CUG triplet repeat, RNA binding protein 1	5 out of 5	−38.352	4.0144 × 10^−5^
MBNL1	muscleblind-like splicing regulator 1	4 out of 5	36.567	7.6664 × 10^−5^
CSTF3	cleavage stimulation factor, 3ʹ pre-RNA, subunit 3	3 out of 5	41.24	1.4073 × 10^−5^
SRSF10	FUS interacting protein (serine/arginine-rich) 1		n.c.	n.c.
GEMIN6	gem (nuclear organelle) associated protein 6		−34.092	0.00018533
HNRNPA2B1	heterogeneous nuclear ribonucleoprotein A2/B1		43.464	6.2483 × 10^−6^
HNRNPH1	heterogeneous nuclear ribonucleoprotein H1 (H)		39.348	2.7932 × 10^−5^
HNRNPH3	heterogeneous nuclear ribonucleoprotein H3 (2H9)		32.941	0.00027984
DDX39B	HLA-B associated transcript 1		n.c.	n.c.
PCBP1	poly(rC) binding protein 1		−35.428	0.0001146
POLR2E	polymerase (RNA) II (DNA directed) polypeptide E, 25 kDa		n.c.	n.c.
POLR2J	polymerase (RNA) II (DNA directed) polypeptide J, 13.3 kDa		−27.037	0.0021827
RPL36AP51	ribosomal protein L36a pseudogene 51		n.c.	n.c.
SRSF1	splicing factor, arginine/serine-rich 1		46.327	2.1799 × 10^−6^
SCAF11	splicing factor, arginine/serine-rich 2, interacting protein		26.307	0.0028008
SRSF3	splicing factor, arginine/serine-rich 3		−42.054	1.0493 × 10^−5^
YTHDC1	YTH domain containing 1		n.c.	n.c.

Combined T stats and *p* values were calculated using the INMEX software. Datasets included in this analysis were: GSE21942, GSE17048, GSE43592, and GSE13732. * The minus sign indicates decreased expression levels in cases with respect to controls. DE: differentially expressed; n.c.: not calculated (not surviving the threshold of significance of 0.05).

As a proof of concept, we hence selected a functional polymorphism (the C to T transition at position chr16:69,605,099; rs12599391) mapping within an intronic splicing enhancer (ISE) and known to influence the inclusion/skipping of an exon of its host gene, *NFAT5* (nuclear factor of activated T-cells 5). In particular, the presence of the C nucleotide was previously demonstrated to disrupt the ISE motif, thus leading to exon-2 skipping [28]. *NFAT5* is an interesting candidate because: (i) it shows a significant exon-specific dysregulation in MS patients compared to controls, as evidenced by Tian and colleagues [10]; and (ii) it might represent a *CELF1/MBNL1* target according to crosslinking-immunoprecipation protocol (CLIP)-seq analysis of mouse cells and tissues [29,30].

**Figure 2:**
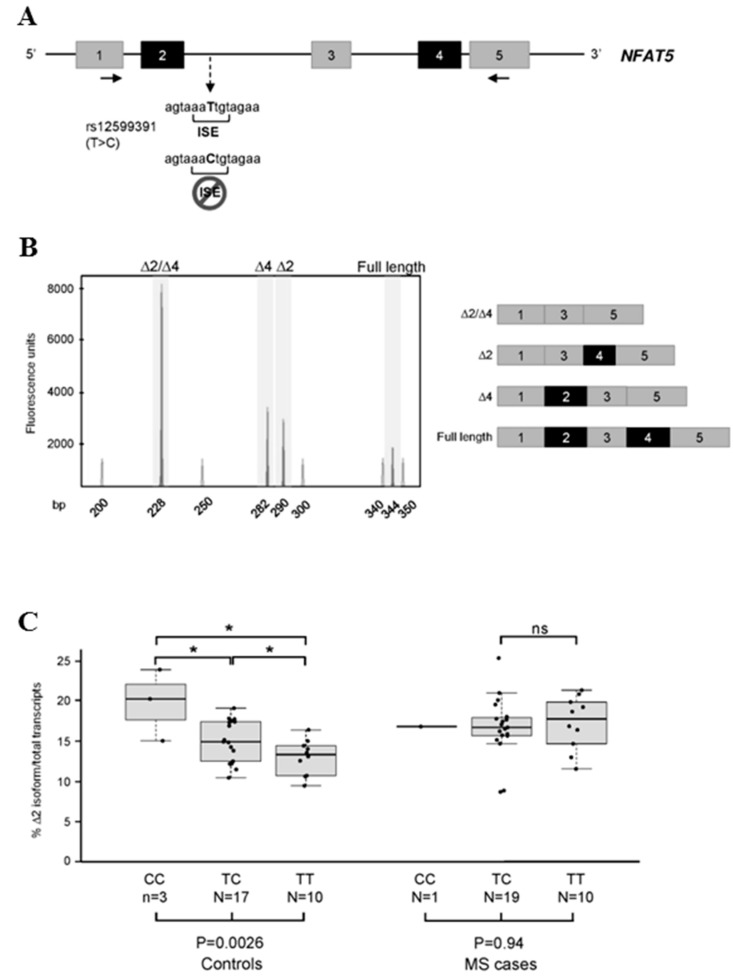
Splicing pattern analysis of the *NFAT5* gene. (**A**) Schematic representation of the *NFAT5* gene between exons 1 and 5. Exons and introns are represented by boxes and lines, and are approximately drawn to scale. Exons indicated in black are those undergoing alternatively skipping (alone or in combination). The positions of the primer couple used for competitive fluorescent RT-PCR experiments are shown by arrows (the forward primer was labeled with the FAM fluorophore). The C > T transition (rs12599391) abolishing the intron-2 ISE element is also indicated; (**B**) Fluorescent RT-PCR products, obtained from the RNA extracted from PBMCs of a control individual (heterozygous CT for the rs12599391 polymorphism), were separated by using capillary electrophoresis. The panel on the left is a close-up view of the GeneMapper window encompassing all peaks corresponding to different isoforms. A schematic representation of the obtained RT-PCR products is also depicted on the right; (**C**) Boxplot diagram showing the percentage of the exon-2-including transcripts among all *NFAT5* mRNAs, according to disease status and by stratifying individuals on the basis of their rs12599391 genotype. Expression levels were measured by fluorescent competitive RT-PCRs. Significance levels of *t*-tests and of the one-way ANOVA analyses, are shown above and below the boxplots, respectively. * *p* < 0.05; ns: not significant.

To specifically detect alternative *NFAT5* isoforms, we developed a fluorescent competitive RT-PCR assay, with primers mapping within exons 1 and 5. RT-PCR assays performed on RNA extracted from PBMCs of five healthy individuals evidenced the existence of at least three different *NFAT5* alternative transcripts, deriving from the skipping of exon 2, 4, or both (Figure 2A,B).

Subsequently, we measured the level of transcripts lacking exon 2 in PBMC RNA samples of our cohort of 30 RR-MS cases and 30 controls, all genotyped for the rs12599391 polymorphism. In general, the level of the exon-2-skipped isoform resulted significantly, though slightly, increased in RR-MS cases *vs.* controls (*p* = 0.02), with the mean percentage of skipping of 16.7% and 14.6% in cases and controls, respectively (data not shown). A remarkable difference was instead evidenced when we compared cases and controls grouped on the basis of their rs12599391 genotype: a clear dependence of exon-2 skipping level upon the C/T genotype was seen in healthy subjects (difference among groups *p* = 0.0026), whereas the distribution observed in RR-MS cases resulted flattened (*p* = 0.94) (Figure 2C). In particular, RR-MS patients showed mean percentages of the exon-2-skipped isoform of 16.62%, 16.56%, and 17.05% for the CC, TC, and TT genotypes, respectively. In controls, the mean percentages resulted 19.73% for CC homozygous individuals and of 15.07% and 12.54% for heterozygotes and TT homozygotes, respectively (Figure 2C).

### 2.5. Discussion

Here, we used publicly available microarray datasets and bioinformatics tools to perform a comprehensive comparative analysis of gene expression profiles in blood-derived cells of a total of 190 RR-MS cases and 182 healthy controls. RR is the principal subtype of MS, representing ~80% of all diagnosed patients, and it is characterized by phases of sudden intensification of symptoms followed by phases of almost complete recovery [31]. In this subtype of MS, inflammatory events play a major role, whereas in the progressive forms of the disease (primary and secondary, ~20% of cases) neurodegeneration is likely prominent. Thus, RR and progressive forms possibly represent different clinical entities [32,33].

Genome-wide microarray analysis of gene expression is actually one of the most powerful tools for the understanding of global changes between normal and pathologic conditions [34]. In this respect, our aim was to verify, in an unsupervised manner, whether the altered expression of genes functionally-related with AS represents a specific signature of MS. In fact, dysregulation of pre-mRNA splicing might act as the convergence point underlying aberrant gene expression changes in MS, thus possibly driving pathogenic mechanisms involved in disease development. In doing this analysis, we considered only datasets including at least 10 RR-MS cases and an equal number of controls. This criterion was adopted to take into account the statistical power issue. Indeed, it is difficult to provide an accurate power estimate for a microarray study. Among others, Wei and colleagues [35] suggested that a sample size of 20 is necessary, at a *p* value of 0.01 and 90% power, to detect a two-fold change in the 75% least variable genes in a microarray study. For this reason, a cut-off value of 10 cases *vs.* 10 controls was considered reasonable. We decided to include for each dataset the top 3000 up/downregulated genes (based on *p* values), independently from the magnitude of the fold change. This approach was chosen to evaluate whether even subtle changes in gene expression levels can have a role “en mass”. Indeed, in a complex disorder like MS, besides few highly dysregulated genes, there could still be potentially thousands of slightly up/downregulated genes, whose effect collectively account for a proportion of the phenotype [36].

Our analysis disclosed two recurrently enriched pathways, belonging to the GO categories of “NADH dehydrogenase complex” and “nuclear mRNA splicing”. Concerning the first category, it is known that mitochondrial dysfunction can mediate neurodegeneration contributing to MS [37,38]. In particular, the NADH dehydrogenase complex (also referred to as Complex I or NADH:ubiquinone oxidoreductase), which is the first enzyme of the mitochondrial respiratory chain [39], was initially demonstrated to have reduced activity in autopsied MS brains [37]. Further sustaining the role of complex I in the pathogenesis of MS, Campbell and colleagues [40] observed large deletions in the mitochondrial genome of neurons microdissected from autopsied MS brains. Moreover, Talla and coworkers [41] successfully tried to suppress neurodegeneration in EAE (experimental autoimmune encephalomyelitis, an established murine model of MS) by overexpressing NDI1, *i.e.*, a subunit of complex I that is able to perform the electron transfer without proton pumping [42]. Hence, our analyses on microarrays studies well support the above-mentioned works in highlighting the involvement of the NADH dehydrogenase complex in MS. However, a confirmation of the dysregulation in expression levels of the transcripts coding for the 44 different polypeptides of this complex is still lacking.

**Figure 3:**
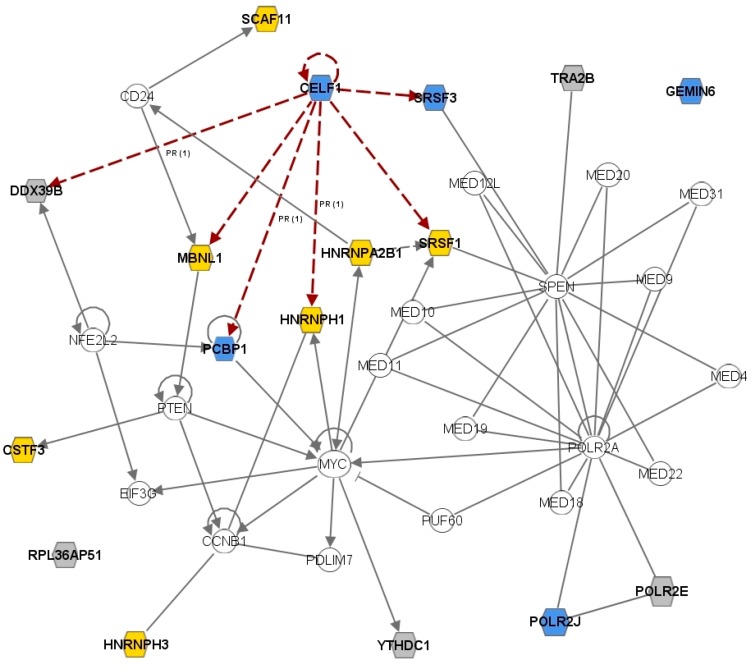
A single network includes most splice regulatory genes dysregulated in MS. The network was generated through the Ingenuity Pathway Analysis software, using as input the list of genes in Table 3. The genes in the original list are represented by hexagonal nodes, whereas those integrated into the computationally-generated network on the basis of the evidence stored in the IPA knowledge memory are indicated as circles. Node color indicates differential expression levels: Yellow: for genes upregulated in MS cases, blue: downregulated in MS cases, and grey: not differentially expressed (Table 3 data). Edges represent gene relationships, with red arrows: indicating *CELF1* targets in resting T cells [45].

As for the “nuclear mRNA splicing” category, our *in-silico* analyses well sustain the hypothesis that initially drove our work, *i.e.*, to verify the contribution of the dysregulation of splicing regulatory proteins to the pathogenesis of MS. The two transcripts that appear to be dysregulated in most datasets are *CELF1* and *MBNL1*: the first was downregulated in RR-MS cases (as also confirmed by our RT-PCR experiments), whereas the second was upregulated (Table 3). Interestingly, the CELF1 and MBNL1 proteins, both binding to CUG repeats, have long been demonstrated to antagonistically control AS during development: MBNL1 promotes adult, whereas CELF1 increases fetal AS patterns for specific exons [43]. An initial genome-wide analysis using CLIP-seq on RNA extracted from the mouse myoblast cell line C2C12, followed by RT-PCR, confirmed that Celf1 and Mbnl1 are able to regulate AS of dozens of transcripts [44]. This same analysis, performed on RNA from various mouse tissues, subsequently revealed that splicing events regulated by Mbnl1 are indeed several hundreds [29]. Even more interestingly for MS pathogenesis, the specific role of CELF1 during human T cell activation was investigated by Beisang and colleagues [45]. In particular, they evidenced a dramatic decrease in the number of CELF1 target transcripts upon T cell activation. Strikingly, among CELF1 targets in resting T cells, we found seven (including CELF1 itself) out of the 17 splicing regulatory genes altered in MS (listed in Table 3). This finding might suggest a functional connection among these 17 genes, as also supported by a pathway analysis that highlighted a single network including most of these splice regulatory genes (Figure 3). Overall these data point to a role for *CELF1* and *MBNL1* as “master regulators” of splicing in mammals, and their dysregulation in MS could hence be regarded as a signal of a generalized, though subtle, dysfunction/impairment of the splicing process in the diseased condition. Of course, our data are too preliminary to draw definite conclusions, and an open question remains whether the observed changes in expression of splicing regulatory proteins (besides *CELF1* and *MBNL1*) are primary effects of the MS pathogenic mechanism or secondary to the disease.

We then decided to verify if the observed generalized splicing alteration might result in the dysregulation of genotype-dependent AS events, as observed for known MS-associated functional polymorphisms [11,12]. We selected the rs12599391 variant, mapping deep in intron 2 of the *NFAT5* gene (3148 nt downstream of exon-2 donor splice site). This variant was shown to influence the inclusion of *NFAT5* exon 2 in the mature transcript in a genotype-dependent manner [28]. We confirmed the expected dependence of exon-2 inclusion on the genotype in healthy individuals, whereas we demonstrated that this sequence-specific control is lost in MS cases (Figure 2C), perhaps as an epiphenomenon of the dysregulation of the splicing process. On the other hand, our results demonstrated that the level of exon-2 inclusion in RR-MS cases is significantly higher than in controls (*p* = 0.02), thus leaving open to discussion whether this could be relevant for MS pathogenesis. The *NFAT5* gene codes for a transcription factor that activates the expression of osmoprotective genes, and is in turn activated by the Th-17-specific PKC-α kinase [46]. In this respect, it is worth noting that the *PRKCA* gene, coding for PKC-α, has repeatedly been associated with MS [12,47]. More interestingly, it has been demonstrated that increased NaCl concentrations significantly boost the induction of human Th-17 cells: High-salt conditions activate the p38/MAPK pathway involving both NFAT5 and serum/glucocorticoid-regulated kinase 1 (SGK1) during cytokine-induced Th-17 polarization, whereas gene silencing/chemical inhibition of *p38/MAPK*, *NFAT5*, or *SGK1* abolishes the high-salt-induced Th-17 development [48]. Th-17 lymphocytes produced in high-salt conditions show a highly-pathogenic phenotype, characterized by the upregulation of pro-inflammatory cytokines such as GM-CSF, TNF-α, and IL-2. Finally, mice raised with a high-salt diet develop a more severe form of EAE, according with an increased CNS-infiltrating and peripherally-induced antigen-specific Th-17 cells [48].

## 3. Experimental Section

### 3.1. Dataset Retrieving and Analyses

The microarray datasets were obtained from the National Center for Biotechnology Information (NCBI) GEO database. The database was searched using “multiple sclerosis”, “blood”, and “Homo sapiens” as keywords. A total of 1727 entries were retrieved; their manual inspection allowed the identification of seven datasets (GSE21942, GSE41848, GSE41849, GSE41890, GSE17048, GSE43592, and GSE13732), respecting the following inclusion criteria: (i) expression data obtained from blood-derived samples; (ii) case-control design; and (iii) data available for at least 10 cases and 10 healthy controls.

Characteristics of selected datasets and their cohort demographics are listed in Table 1.

The GEO2R web application [49,50], available at GEO repository, was used for identifying differentially expressed genes in the selected datasets. GEO2R implements established Bioconductor R packages [51,52] and provides results as a table of genes ordered by significance (*p* value). The seven datasets were analyzed separately by comparing cases *vs.* controls. For each study, the resulting top 3000 up/downregulated genes were considered for further analyses, independently from the magnitude of the fold change and of the corresponding significance level. To search for enriched functionally-related gene groups, the seven lists of 3000 dysregulated genes were submitted to DAVID v6.7, using the Functional annotation clustering tool with default annotation categories and highest stringency [27]. This procedure allows the identification of functionally-related genes ranked on the basis of their enrichment score (defined in DAVID as the geometric mean of all the enrichment *p*-values for each annotation term associated with the gene members in the group) [27]. Only groups of functionally-related genes showing an enrichment score above 1.3 (corresponding to *p* values below 0.05) were hence considered. Annotation clusters enriched in “RNA splicing” terms were used to retrieve the list of recurrently altered genes.

To corroborate GEO2R results, a meta-analysis using the Integrative Meta-analysis of Expression Data (INMEX) program [53] was also carried out on four eligible datasets (GSE21942, GSE17048, GSE43592, and GSE13732). The remaining three datasets were not included since they were collected using the Affymetrix Human Exon 1.0 ST Array, a platform/data format not allowed by INMEX. Datasets were uploaded to INMEX, processed, annotated, and checked for their integrity. The meta-analysis was performed using the “Combining *p*-values” option, based on the Fisher’s method [−2*∑Log(p)] [53], setting the threshold for significance at 0.05. Functional gene ontology analysis of the differentially expressed genes was hence performed using the “Molecular function” option with a *p* value threshold of 0.0005.

Finally, a list of genes, belonging to the GO “RNA splicing” category and resulting differentially dysregulated in at least three datasets, was analyzed by using the QIAGEN’s Ingenuity Pathway Analysis (IPA) software (QIAGEN, Redwood City, CA, USA).

### 3.2. MS Cases and Healthy Controls

This study was approved by local Ethical Committees and was conducted according to the Declaration of Helsinki and to the Italian legislation on sensible data recording. All the recruited subjects signed an informed consent.

We enrolled 30 unrelated RR MS patients and 30 healthy subjects. To avoid possible confounding effects, we decided to specifically focus on RR-MS cases in remitting phase, *i.e.*, patients who had not received any immunomodulatory therapy within the month prior to blood withdrawal. To avoid possible confounder effects due to diurnal variation in immune function, all samples were collected between 08:00 and 11:00. Among patients (mean age 43 ± 7 years), 22 were females.

Controls were matched with cases in terms of gender and age. All individuals were Caucasians and coming from Northern Italy.

### 3.3. RNA and DNA Samples

PBMCs were isolated from blood by centrifugation on a Lympholyte Cell separation medium (Cederlane Laboratories Limited: Ontario, Canada) gradient. All blood samples were processed immediately after withdrawal. Total RNA was isolated using the Eurozol kit (EuroClone: Wetherby, UK). RNA concentration was determined using the NanoDrop ND-1000 spectrophotometer (NanoDrop Technologies: Wilmington, DE, USA). RNA quality was assessed on an Agilent Bioanalyzer 2100 using the Agilent RNA 6000 Nano Assay kit (Agilent Technologies: Santa Clara, CA, USA).

Genomic DNA extraction was performed from peripheral blood of the same patients/controls by using the Microlab STAR Liquid Handler (Hamilton: Bonaduz, Switzerland) integrated with Chemagen automated DNA extraction system (Chemagen AG: Baesweiler, Germany). DNA samples were quantified by using a PicoGreen assay (Thermo Fisher Scientific: Waltham, MA, USA) and the microplate reader Wallac 1420 VICTOR3 V (Perkin Elmer: Waltham, MA, USA).

### 3.4. Semi-Quantitative Real-Time RT-PCR and Competitive Fluorescent RT-PCR

Random nonamers and the Superscript-III Reverse Transcriptase (Thermo Fisher Scientific) were used to perform first-strand cDNA synthesis starting from 1 µg of total RNA, according to the manufacturer’s instructions. Of a total of 20 µL of the reverse-transcription (RT) reaction, 1 µL was used as template for subsequent amplifications.

Semi-quantitative real-time RT-PCRs for the quantitation of the CUGBP Elav-like family member 1 (*CELF1*) transcript were carried out using the FastStart SYBR Green Master mix (Roche Applied Science: Basel, Switzerland). Reactions were performed at least in triplicate on a LightCycler 480 (Roche Applied Science), following a touchdown thermal protocol. Expression levels were normalized using two housekeeping genes (hydroxymethylbilane synthase [*HMBS*]; β-actin [*ACTB*]). Data were analyzed using the GeNorm software [54].

To specifically quantify the relative amount of alternatively-spliced isoforms of the *NFAT5* gene, we took advantage of their length differences, and performed competitive RT-PCRs under standard conditions by using a 6-FAM-labeled primer (Sigma: St. Louis, MO, USA). Amplified fragments were separated by capillary electrophoresis on an ABI-3130XL Genetic Analyzer (Thermo Fisher Scientific) and quantitated by the GeneMapper v4.0 software (Thermo Fisher Scientific). The sum of all fluorescence peak areas in a single run was set equal to 100%, and the relative quantity of each transcript expressed as a fraction of the total. In all cases, a preliminary assessment of amplification efficiency was performed by real-time PCR (amplicons showed identical amplification efficiencies) (Roche Applied Science).

Primer couples used in these assays are listed in Table S5.

### 3.5. Genotyping of the rs12599391 Polymorphism in the NFAT5 Gene

Genotyping of the rs12599391 polymorphism was performed by direct DNA sequencing. A PCR primer couple was designed in order to amplify the genomic region containing the polymorphic variant (Table S5). Standard PCRs were performed on 20 ng of genomic DNA in a 25-μL final volume using the FastStart Taq DNA Polymerase (Roche Applied Science). Direct sequencing of PCR products was performed by the fluorescent dideoxy terminator method (BigDye Terminator Cycle Sequencing Ready Reaction Kit v1.1; Thermo Fisher Scientific), and analyzed by using an ABI-3130XL Genetic Analyzer (Thermo Fisher Scientific). The Variant Reporter software was used for variant calling (Thermo Fisher Scientific).

### 3.6. Web Resources

The URLs for data mining and analyses presented herein detailed in the “References and Notes” section [52,55,56,57,58,59,60].

## 4. Conclusions

In conclusion, our study provides for the first time a robust evidence that dysregulation in the AS process can be relevant for MS pathogenesis, pointing to RNA processing as a possible pathway to be targeted for MS treatment. In addition, our data suggest caution in interpreting conflicting results obtained so far on the use of small molecules in the treatment of the disease [61,62,63]. For instance, resveratrol (a modulator of RNA-processing factor levels) showed a neuroprotective effect in an EAE mouse model [62], whereas it exacerbated demyelination and inflammation in the absence of neuroprotection in the CNS of two models of experimentally-induced encephalomyelitis [63]. This may be due, at least in part, to differences in antigens and animal strains [63]. A better knowledge of the actual molecular targets of resveratrol will be needed to predict its global effect on splicing and subsequent phenotypic outcomes.

## Supplementary Materials

Supplementary materials can be found at http://www.mdpi.com/1422-0067/16/10/23463/s1.
