# Supplementary material for: Meta-Analysis of Multiple Sclerosis Microarray Data Reveals Dysregulation in RNA Splicing Regulatory Genes
Source: Int J Mol Sci. 2015 Sep 30;16(10):23463–81. doi: 10.3390/ijms161023463 (PMC4632709; doi:10.3390/ijms161023463)
Supplement: Supplementary file 1 [file ijms-16-23463-s001.pdf]

Supplementary Information

Table S1. *p*-Value ranges of gene lists submitted to DAVID.

Dataset (Accession Number)	Array Type	MS Cases/Controls	*p* Value of the 1st Ranked Gene *	*p* Value of the 3000th Ranked Gene *
GSE21942	Affymetrix Human Genome U133 Plus 2.0 Array	10/15	7.05×10^{-14}	5.97×10^{-5}
GSE41848	Affymetrix Human Exon 1.0 ST Array	54/38 (discovery dataset)	1.76×10^{-7}	3.44×10^{-2}
GSE41849	Affymetrix Human Exon 1.0 ST Array	21/22 (replication dataset)	7.15×10^{-5}	0.244
GSE41890	Affymetrix Human Gene 1.0 ST Array	22/24	2.32×10^{-4}	0.149
GSE17048	Illumina HumanHT-12 V3.0 Expression BeadChip	36/45	3.61×10^{-7}	6.07×10^{-2}
GSE43592	Affymetrix Human Genome U133 Plus 2.0 Array	10/10	2.51×10^{-7}	9.65×10^{-3}
GSE13732	Affymetrix Human Genome U133 Plus 2.0 Array	37/28	2.02×10^{-25}	1.62×10^{-7}

* Uncorrected *p* Values are presented.

Table S2. (**A**–**H**) Main results of the Database for Annotation, Visualization and Integrated Discovery (DAVID) analysis.

(**A**)

**GSE21942**				
Only the first 10 annotation clusters are reported (they are all significantly enriched; Enrichment score > 1.3, $p < 0.05$)				
Annotation Cluster 1	Enrichment Score: 12	Count	*p* Value	Benjamini
GOTERM_BP_FAT	nuclear mRNA splicing, via spliceosome	54	1.0×10^{-12}	2.7×10^{-10}
GOTERM_BP_FAT	RNA splicing, via transesterification reactions with bulged adenosine as nucl	54	1.0×10^{-12}	2.7×10^{-10}
GOTERM_BP_FAT	RNA splicing, via transesterification reactions	54	1.0×10^{-12}	2.7×10^{-10}
Annotation Cluster 2	Enrichment Score: 5.78	Count	*p* Value	Benjamini
GOTERM_BP_FAT	regulation of apoptosis	147	1.2×10^{-6}	1.3×10^{-4}
GOTERM_BP_FAT	regulation of cell death	148	1.7×10^{-6}	1.7×10^{-4}
GOTERM_BP_FAT	regulation of programmed cell death	147	2.2×10^{-6}	2.2×10^{-4}
Annotation Cluster 3	Enrichment Score: 3.86	Count	*p* Value	Benjamini
GOTERM_BP_FAT	negative regulation of apoptosis	70	9.9×10^{-5}	4.7×10^{-3}
GOTERM_BP_FAT	negative regulation of programmed cell death	70	1.5×10^{-4}	6.5×10^{-3}
GOTERM_BP_FAT	negative regulation of cell death	70	1.7×10^{-4}	7.0×10^{-3}
Annotation Cluster 4	Enrichment Score: 3.74	Count	*p* Value	Benjamini
GOTERM_BP_FAT	mitochondrial ATP synthesis coupled electron transport	19	9.8×10^{-5}	4.8×10^{-3}
GOTERM_BP_FAT	ATP synthesis coupled electron transport	19	9.8×10^{-5}	4.8×10^{-3}
GOTERM_BP_FAT	respiratory electron transport chain	19	6.3×10^{-4}	2.1×10^{-2}

Table S2. *Cont.*

(**A**)

**GSE21942**				
Only the first 10 annotation clusters are reported (they are all significantly enriched; Enrichment score > 1.3, $p < 0.05$)				
Annotation Cluster 5	Enrichment Score: 3.44	Count	*p* Value	Benjamini
UP_SEQ_FEATURE	repeat:WD 1	52	2.6×10^{-4}	6.1×10^{-2}
UP_SEQ_FEATURE	repeat:WD 2	52	2.6×10^{-4}	6.1×10^{-2}
SP_PIR_KEYWORDS	wd repeat	52	2.7×10^{-4}	4.0×10^{-3}
INTERPRO	WD40 repeat, conserved site	52	9.4×10^{-4}	1.2×10^{-1}
Annotation Cluster 6	Enrichment Score: 3.42	Count	*p* Value	Benjamini
INTERPRO	Cullin, N-terminal region	7	3.8×10^{-4}	8.6×10^{-2}
INTERPRO	Cullin, N-terminal	7	3.8×10^{-4}	8.6×10^{-2}
INTERPRO	Cullin, conserved site	7	3.8×10^{-4}	8.6×10^{-2}
Annotation Cluster 7	Enrichment Score: 3.05	Count	*p* Value	Benjamini
UP_SEQ_FEATURE	domain:LisH	11	6.7×10^{-4}	1.0×10^{-1}
INTERPRO	LisH dimerisation motif	11	9.5×10^{-4}	1.1×10^{-1}
SMART	LisH	11	1.1×10^{-3}	6.5×10^{-2}
Annotation Cluster 8	Enrichment Score: 3	Count	*p* Value	Benjamini
GOTERM_CC_FAT	NADH dehydrogenase complex	14	7.8×10^{-4}	9.1×10^{-3}
GOTERM_CC_FAT	Respiratory chain complex I	14	7.8×10^{-4}	9.1×10^{-3}
GOTERM_CC_FAT	Mitochondrial respiratory chain complex I	14	7.8×10^{-4}	9.1×10^{-3}
GOTERM_MF_FAT	NADH dehydrogenase activity	14	1.3×10^{-3}	4.9×10^{-2}
GOTERM_MF_FAT	NADH dehydrogenase (quinone) activity	14	1.3×10^{-3}	4.9×10^{-2}
GOTERM_MF_FAT	NADH dehydrogenase (ubiquinone) activity	14	1.3×10^{-3}	4.9×10^{-2}
Annotation Cluster 9	Enrichment Score: 2.95	Count	*p* Value	Benjamini
UP_SEQ_FEATURE	Cross-link:Glycyl lysine isopeptide (Lys–Gly) (interchain with G–Cter in NEDD	6	3.3×10^{-4}	6.3×10^{-2}
INTERPRO	Cullin homology	6	2.0×10^{-3}	1.7×10^{-1}
SMART	Cullin	6	2.2×10^{-3}	8.3×10^{-2}
Annotation Cluster 10	Enrichment Score: 2.93	Count	*p* Value	Benjamini
GOTERM_BP_FAT	Establishment of RNA	25	6.9×10^{-4}	2.3×10^{-2}
GOTERM_BP_FAT	localization RNA transport	25	6.9×10^{-4}	2.3×10^{-2}
GOTERM_BP_FAT	nucleic acid transport	25	6.9×10^{-4}	2.3×10^{-2}
GOTERM_BP_FAT	RNA localization	25	1.1×10^{-3}	3.4×10^{-2}
GOTERM_BP_FAT	nucleobase, nucleoside, nucleotide and nucleic acid transport	25	6.0×10^{-3}	1.3×10^{-1}

(**B**)

**GSE41848**				
Only the first 10 annotation clusters are reported (they are all significantly enriched; Enrichment score > 1.3, $p < 0.05$)				
Annotation Cluster 1	Enrichment Score: 4.2	Count	*p* Value	Benjamini
SMART	PI3Kc	11	4.6×10^{-5}	6.3×10^{-3}
UP_SEQ_FEATURE	domain:PI3K/PI4K	11	6.2×10^{-5}	5.9×10^{-2}
INTERPRO	Phosphatidylinositol 3- and 4-kinase, catalytic	11	7.6×10^{-5}	2.0×10^{-1}
INTERPRO	Phosphatidylinositol 3- and 4-kinase, conserved site	11	7.6×10^{-5}	2.0×10^{-1}

Table S2. *Cont.*

(**B**)

GSE41848				
Only the first 10 annotation clusters are reported (they are all significantly enriched; Enrichment score > 1.3, $p < 0.05$)				
Annotation Cluster 2	Enrichment Score: 3.81	Count	*p* Value	Benjamini
SMART	WD40	63	3.3×10^{-5}	6.8×10^{-3}
INTERPRO	WD40 repeat	63	1.9×10^{-4}	8.8×10^{-2}
UP_SEQ_FEATURE	repeat:WD 2	63	2.4×10^{-4}	1.3×10^{-1}
UP_SEQ_FEATURE	repeat:WD 1	63	2.4×10^{-4}	1.3×10^{-1}
SP_PIR_KEYWORDS	wd repeat	63	2.5×10^{-4}	5.1×10^{-3}
Annotation Cluster 3	Enrichment Score: 3.43	Count	*p* Value	Benjamini
UP_SEQ_FEATURE	domain:FAT	6	3.5×10^{-4}	1.5×10^{-1}
UP_SEQ_FEATURE	domain:FATC	6	3.5×10^{-4}	1.5×10^{-1}
INTERPRO	PIK-related kinase	6	3.9×10^{-4}	9.1×10^{-2}
INTERPRO	PIK-related kinase, FATC	6	3.9×10^{-4}	9.1×10^{-2}
Annotation Cluster 4	Enrichment Score: 3.13	Count	*p* Value	Benjamini
UP_SEQ_FEATURE	domain:JmjC	13	6.1×10^{-4}	1.9×10^{-1}
SMART	JmjC	13	6.4×10^{-4}	4.3×10^{-2}
INTERPRO	Transcription factor jumonji/ aspartyl beta-hydroxylase	13	1.1×10^{-3}	2.0×10^{-1}
Annotation Cluster 5	Enrichment Score: 3.04	Count	*p* Value	Benjamini
SP_PIR_KEYWORDS	bromodomain	15	7.6×10^{-4}	1.3×10^{-2}
SMART	BROMO	15	7.6×10^{-4}	4.4×10^{-2}
INTERPRO	Bromodomain	15	1.4×10^{-3}	2.0×10^{-1}
Annotation Cluster 6	Enrichment Score: 2.97	Count	*p* Value	Benjamini
GOTERM_CC_FAT	mitochondrial respiratory chain complex I	16	9.4×10^{-4}	1.4×10^{-2}
GOTERM_CC_FAT	NADH dehydrogenase complex	16	9.4×10^{-4}	1.4×10^{-2}
GOTERM_CC_FAT	respiratory chain complex I	16	9.4×10^{-4}	1.4×10^{-2}
GOTERM_MF_FAT	NADH dehydrogenase activity	16	1.2×10^{-3}	5.6×10^{-2}
GOTERM_MF_FAT	NADH dehydrogenase (ubiquinone) activity	16	1.2×10^{-3}	5.6×10^{-2}
GOTERM_MF_FAT	NADH dehydrogenase (quinone) activity	16	1.2×10^{-3}	5.6×10^{-2}
Annotation Cluster 7	Enrichment Score: 2.67	Count	*p* Value	Benjamini
GOTERM_BP_FAT	nuclear mRNA splicing, via spliceosome	39	2.2×10^{-3}	9.8×10^{-2}
GOTERM_BP_FAT	RNA splicing, via transesterification reactions	39	2.2×10^{-3}	9.8×10^{-2}
GOTERM_BP_FAT	RNA splicing, via transesterification reactions with bulged adenosine as nucleophile	39	2.2×10^{-3}	9.8×10^{-2}
Annotation Cluster 8	Enrichment Score: 2.33	Count	*p* Value	Benjamini
GOTERM_BP_FAT	ATP synthesis coupled proton transport	15	2.1×10^{-3}	9.9×10^{-2}
GOTERM_BP_FAT	energy coupled proton transport, down electrochemical gradient	15	2.1×10^{-3}	9.9×10^{-2}
SP_PIR_KEYWORDS	Hydrogen ion transport	15	6.8×10^{-3}	7.9×10^{-2}
GOTERM_BP_FAT	ion transmembrane transport	15	1.6×10^{-2}	3.5×10^{-1}

Table S2. *Cont.*

(**B**)

**GSE41848**				
Only the first 10 annotation clusters are reported (they are all significantly enriched; Enrichment score > 1.3, $p < 0.05$)				
Annotation Cluster 9	Enrichment Score: 2.29	Count	*p* Value	Benjamini
GOTERM_BP_FAT	Golgi transport vesicle coating	7	1.8×10^{-3}	8.8×10^{-2}
GOTERM_BP_FAT	Golgi vesicle budding	7	1.8×10^{-3}	8.8×10^{-2}
GOTERM_BP_FAT	COPI coating of Golgi vesicle	7	1.8×10^{-3}	8.8×10^{-2}
GOTERM_BP_FAT	Vesicle targeting, to, from or within Golgi	7	5.8×10^{-3}	2.0×10^{-1}
GOTERM_BP_FAT	Vesicle targeting	7	1.2×10^{-1}	8.3×10^{-1}
Annotation Cluster 10	Enrichment Score: 2.22	Count	*p* Value	Benjamini
SP_PIR_KEYWORDS	Threonine protease	9	4.8×10^{-3}	6.1×10^{-2}
GOTERM_MF_FAT	Threonine type endopeptidase activity	9	6.8×10^{-3}	2.0×10^{-1}
GOTERM_MF_FAT	Threonine type peptidase activity	9	6.8×10^{-3}	2.0×10^{-1}

(**C**)

**GSE41849**				
Only the 9 annotation clusters that resulted significantly enriched (Enrichment score > 1.3, $p < 0.05$) are reported				
Annotation Cluster 1	Enrichment Score: 5.52	Count	*p* Value	Benjamini
GOTERM_MF_FAT	NADH dehydrogenase (ubiquinone) activity	20	1.7×10^{-6}	1.2×10^{-3}
GOTERM_MF_FAT	NADH dehydrogenase (quinone) activity	20	1.7×10^{-6}	1.2×10^{-3}
GOTERM_MF_FAT	NADH dehydrogenase activity	20	1.7×10^{-6}	1.2×10^{-3}
GOTERM_MF_FAT	oxidoreductase activity, acting on NADH or NADPH, quinone or similar compound as acceptor	20	1.7×10^{-5}	6.0×10^{-3}
Annotation Cluster 2	Enrichment Score: 5.1	Count	*p* Value	Benjamini
GOTERM_CC_FAT	NADH dehydrogenase complex	19	7.9×10^{-6}	2.2×10^{-4}
GOTERM_CC_FAT	mitochondrial respiratory chain complex I	19	7.9×10^{-6}	2.2×10^{-4}
GOTERM_CC_FAT	respiratory chain complex I	19	7.9×10^{-6}	2.2×10^{-4}
Annotation Cluster 3	Enrichment Score: 1.92	Count	*p* Value	Benjamini
GOTERM_MF_FAT	oxidoreductase activity, acting on heme group of donors, oxygen as acceptor	10	1.2×10^{-2}	4.9×10^{-1}
GOTERM_MF_FAT	oxidoreductase activity, acting on heme group of donors	10	1.2×10^{-2}	4.9×10^{-1}
GOTERM_MF_FAT	Cytochrome-c oxidase activity	10	1.2×10^{-2}	4.9×10^{-1}
GOTERM_MF_FAT	Heme-copper terminal oxidase activity	10	1.2×10^{-2}	4.9×10^{-1}
Annotation Cluster 4	Enrichment Score: 1.82	Count	*p* Value	Benjamini
INTERPRO	Ribosomal protein S27a	4	9.8×10^{-3}	9.9×10^{-1}
INTERPRO	Ribosomal protein L40e	4	9.8×10^{-3}	9.9×10^{-1}
UP_SEQ_FEATURE	chain:40S ribosomal protein S27a	4	9.9×10^{-3}	1.0
UP_SEQ_FEATURE	chain:Ubiquitin	4	9.9×10^{-3}	1.0
UP_SEQ_FEATURE	site:Essential for function	4	9.9×10^{-3}	1.0
UP_SEQ_FEATURE	compositionally biased region:Lys-rich (highly basic)	4	9.9×10^{-3}	1.0
PIR_SUPERFAMILY	PIRSF002042:polyubiquitin 3	4	1.3×10^{-2}	1.0
PIR_SUPERFAMILY	PIRSF038431:ribosomal protein S27Ae	4	1.3×10^{-2}	1.0

Table S2. *Cont.*

(**C**)

**GSE41849**				
Only the 9 annotation clusters that resulted significantly enriched (Enrichment score > 1.3, $p < 0.05$) are reported				
SP_PIR_KEYWORDS	polyprotein	4	2.2×10^{-2}	3.8×10^{-1}
UP_SEQ_FEATURE	binding site:Activating enzyme	4	2.2×10^{-2}	1.0
GOTERM_BP_FAT	Long-term strengthening of neuromuscular junction	4	2.5×10^{-2}	9.1×10^{-1}
GOTERM_BP_FAT	regulation of synaptic growth at neuromuscular junction	4	7.0×10^{-2}	9.6×10^{-1}
Annotation Cluster 5	Enrichment Score: 1.76	Count	*p* Value	Benjamini
GOTERM_BP_FAT	positive regulation of ubiquitin-protein ligase activity during mitotic cell cycle	19	8.1×10^{-3}	7.4×10^{-1}
GOTERM_BP_FAT	positive regulation of ubiquitin-protein ligase activity	19	1.1×10^{-2}	8.2×10^{-1}
GOTERM_BP_FAT	regulation of ubiquitin-protein ligase activity during mitotic cell cycle	19	1.3×10^{-2}	8.4×10^{-1}
GOTERM_BP_FAT	positive regulation of ligase activity	19	1.7×10^{-2}	8.7×10^{-1}
GOTERM_BP_FAT	regulation of ubiquitin-protein ligase activity	19	3.2×10^{-2}	9.2×10^{-1}
GOTERM_BP_FAT	regulation of ligase activity	19	4.5×10^{-2}	9.3×10^{-1}
Annotation Cluster 6	Enrichment Score: 1.56	Count	*p* Value	Benjamini
GOTERM_BP_FAT	negative regulation of ubiquitin-protein ligase activity during mitotic cell cycle	17	2.4×10^{-2}	9.1×10^{-1}
GOTERM_BP_FAT	Anaphase-promoting complex-dependent proteasomal ubiquitin-dependent protein catabolic process	17	2.4×10^{-2}	9.1×10^{-1}
GOTERM_BP_FAT	negative regulation of ligase activity	17	3.1×10^{-2}	9.2×10^{-1}
GOTERM_BP_FAT	negative regulation of ubiquitin-protein ligase activity	17	3.1×10^{-2}	9.2×10^{-1}
Annotation Cluster 7	Enrichment Score: 1.51	Count	*p* Value	Benjamini
INTERPRO	Cytochrome b561, eukaryote	4	2.2×10^{-2}	9.9×10^{-1}
PIR_SUPERFAMILY	PIRSF016525:cytochrome b561	4	2.9×10^{-2}	1.0
SMART	B561	4	2.9×10^{-2}	9.8×10^{-1}
INTERPRO	Cytochrome b561/ferric reductase transmembrane	4	3.9×10^{-2}	1.0
UP_SEQ_FEATURE	domain:Cytochrome b561	4	4.0×10^{-2}	1.0
Annotation Cluster 8	Enrichment Score: 1.4	Count	*p* Value	Benjamini
GOTERM_MF_FAT	NAPE-specific phospholipase D activity	4	2.2×10^{-2}	6.7×10^{-1}
SMART	PLDc	4	4.6×10^{-2}	9.2×10^{-1}
INTERPRO	Phospholipase D/Transphosphatidylase	4	6.2×10^{-2}	1.0
Annotation Cluster 9	Enrichment Score: 1.35	Count	*p* Value	Benjamini
PIR_SUPERFAMILY	PIRSF005651:membrane protease subunits, stomatin/prohibitin homologs	5	1.3×10^{-2}	9.9×10^{-1}
SMART	PHB	5	6.8×10^{-2}	9.1×10^{-1}
INTERPRO	Band 7 protein	5	9.7×10^{-2}	1.0

Table S2. *Cont.*

(**D**)

GSE41890				
Only the first 10 annotation clusters are reported (they are all significantly enriched; Enrichment score > 1.3, $p < 0.05$)				
Annotation Cluster 1	Enrichment Score: 5.1	Count	*p* Value	Benjamini
GOTERM_BP_FAT	nuclear division	45	5.4×10^{-6}	4.1×10^{-3}
GOTERM_BP_FAT	mitosis	45	5.4×10^{-6}	4.1×10^{-3}
GOTERM_BP_FAT	M phase of mitotic cell cycle	45	8.7×10^{-6}	5.5×10^{-3}
GOTERM_BP_FAT	organelle fission	45	1.6×10^{-5}	7.3×10^{-3}
Annotation Cluster 2	Enrichment Score: 3.15	Count	*p* Value	Benjamini
UP_SEQ_FEATURE	domain: C-type lectin	19	3.6×10^{-4}	7.6×10^{-1}
INTERPRO	*C*-type lectin	19	6.7×10^{-4}	3.5×10^{-1}
INTERPRO	*C*-type lectin, conserved site	19	6.7×10^{-4}	3.5×10^{-1}
SMART	CLECT	19	1.5×10^{-3}	4.0×10^{-1}
Annotation Cluster 3	Enrichment Score: 2.64	Count	*p* Value	Benjamini
UP_SEQ_FEATURE	region of interest:Beta	6	1.3×10^{-3}	8.2×10^{-1}
UP_SEQ_FEATURE	region of interest:Alpha	6	1.3×10^{-3}	8.2×10^{-1}
PIR_SUPERFAMILY	PIRSF002564:metallothionein	6	2.0×10^{-3}	7.5×10^{-1}
UP_SEQ_FEATURE	metal ion-binding site:Divalent metal cation; cluster A	6	2.2×10^{-3}	8.2×10^{-1}
UP_SEQ_FEATURE	metal ion-binding site:Divalent metal cation; cluster B	6	2.2×10^{-3}	8.2×10^{-1}
INTERPRO	Metallothionein, vertebrate, metal binding site	6	2.3×10^{-3}	6.7×10^{-1}
SP_PIR_KEYWORDS	Metal-thiolate cluster	6	3.4×10^{-3}	1.1×10^{-1}
INTERPRO	Metallothionein, vertebrate	6	3.7×10^{-3}	6.3×10^{-1}
INTERPRO	Metallothionein superfamily, eukaryotic	6	3.7×10^{-3}	6.3×10^{-1}
Annotation Cluster 4	Enrichment Score: 2.19	Count	*p* Value	Benjamini
INTERPRO	Kinesin, motor region	11	5.6×10^{-3}	6.6×10^{-1}
INTERPRO	Kinesin, motor region, conserved site	11	5.6×10^{-3}	6.6×10^{-1}
UP_SEQ_FEATURE	domain:Kinesin-motor	11	6.1×10^{-3}	9.3×10^{-1}
SMART	KISc	11	8.9×10^{-3}	6.4×10^{-1}
Annotation Cluster 5	Enrichment Score: 2.01	Count	*p* Value	Benjamini
INTERPRO	Small chemokine, interleukin-8-like	11	4.6×10^{-3}	6.3×10^{-1}
SMART	SCY	11	7.5×10^{-3}	7.2×10^{-1}
GOTERM_MF_FAT	chemokine activity	11	1.3×10^{-2}	8.9×10^{-1}
GOTERM_MF_FAT	chemokine receptor binding	11	2.0×10^{-2}	8.4×10^{-1}
Annotation Cluster 6	Enrichment Score: 1.98	Count	*p* Value	Benjamini
UP_SEQ_FEATURE	domain:MIF4G	5	6.9×10^{-3}	9.4×10^{-1}
INTERPRO	MIF4G-like, type 3	5	1.1×10^{-2}	8.6×10^{-1}
INTERPRO	MIF4-like, type 1/2/3	5	1.1×10^{-2}	8.6×10^{-1}
SMART	MIF4G	5	1.4×10^{-2}	7.1×10^{-1}
Annotation Cluster 7	Enrichment Score: 1.91	Count	*p* Value	Benjamini
UP_SEQ_FEATURE	domain:MI	4	7.4×10^{-3}	9.3×10^{-1}
INTERPRO	Initiation factor Eif-4 gamma, MA3	4	1.4×10^{-2}	9.0×10^{-1}
SMART	MA3	4	1.7×10^{-2}	7.0×10^{-1}

Table S2. *Cont.*

(**D**)

**GSE41890**				
Only the first 10 annotation clusters are reported (they are all significantly enriched; Enrichment score > 1.3, $p < 0.05$)				
Annotation Cluster 8	Enrichment Score: 1.76	Count	*p* Value	Benjamini
UP_SEQ_FEATURE	domain:PNT	5	1.6×10^{-2}	9.9×10^{-1}
INTERPRO	Sterile alpha motif/pointed	5	1.6×10^{-2}	9.1×10^{-1}
SMART	SAM_PNT	5	2.1×10^{-2}	7.0×10^{-1}
Annotation Cluster 9	Enrichment Score: 1.72	Count	*p* Value	Benjamini
UP_SEQ_FEATURE	domain:PARP catalytic	7	3.6×10^{-3}	8.3×10^{-1}
INTERPRO	Poly(ADP-ribose) polymerase, catalytic region	7	3.8×10^{-3}	6.0×10^{-1}
GOTERM_MF_FAT	NAD + ADP-ribosyltransferase activity	7	3.2×10^{-2}	8.7×10^{-1}
GOTERM_MF_FAT	transferase activity, transferring pentosyl groups	7	3.0×10^{-1}	9.9×10^{-1}
Annotation Cluster 10	Enrichment Score: 1.47	Count	*p* Value	Benjamini
PIR_SUPERFAMILY	PIRSF005552:guanine nucleotide-binding protein 1	4	2.1×10^{-2}	9.8×10^{-1}
INTERPRO	Guanylate-binding protein, C-terminal	4	2.3×10^{-2}	9.4×10^{-1}
INTERPRO	Guanylate-binding protein, N-terminal	4	8.2×10^{-2}	9.8×10^{-1}

(**E**)

**GSE17048**				
Only the 10 annotation clusters that resulted significantly enriched (Enrichment score > 1.3, $p < 0.05$) are reported				
Annotation Cluster 1	Enrichment Score: 2.41	Count	*p* Value	Benjamini
SMART	PX	12	3.4×10^{-3}	6.9×10^{-1}
UP_SEQ_FEATURE	domain:PX	12	3.6×10^{-3}	9.9×10^{-1}
INTERPRO	Phox-like	12	4.9×10^{-3}	1.0
Annotation Cluster 2	Enrichment Score: 2.41	Count	*p* Value	Benjamini
GOTERM_CC_FAT	Proton-transporting two-sector ATPase complex	13	8.0×10^{-4}	1.9×10^{-2}
SP_PIR_KEYWORDS	Hydrogen ion transport	13	1.2×10^{-3}	7.2×10^{-2}
GOTERM_BP_FAT	proton transport	13	1.4×10^{-2}	6.0×10^{-1}
GOTERM_BP_FAT	hydrogen transport	13	1.7×10^{-2}	6.5×10^{-1}
Annotation Cluster 3	Enrichment Score: 2.17	Count	*p* Value	Benjamini
GOTERM_BP_FAT	ATP synthesis coupled proton transport	11	4.2×10^{-3}	4.7×10^{-1}
GOTERM_BP_FAT	energy coupled proton transport, down electrochemical gradient	11	4.2×10^{-3}	4.7×10^{-1}
GOTERM_BP_FAT	ion transmembrane transport	11	1.8×10^{-2}	6.6×10^{-1}
Annotation Cluster 4	Enrichment Score: 2.16	Count	*p* Value	Benjamini
GOTERM_BP_FAT	nuclear mRNA splicing, via spliceosome	26	6.9×10^{-3}	4.9×10^{-1}
GOTERM_BP_FAT	RNA splicing, via transesterification reactions with bulged adenosine as nucleophile	26	6.9×10^{-3}	4.9×10^{-1}
GOTERM_BP_FAT	RNA splicing, via transesterification reactions	26	6.9×10^{-3}	4.9×10^{-1}
Annotation Cluster 5	Enrichment Score: 2.13	Count	*p* Value	Benjamini
GOTERM_BP_FAT	positive regulation of ubiquitin-protein ligase activity during mitotic cell cycle	15	5.3×10^{-3}	4.6×10^{-1}
GOTERM_BP_FAT	positive regulation of ubiquitin-protein ligase activity	15	6.9×10^{-3}	4.9×10^{-1}

Table S2. *Cont.*

(**E**)

**GSE17048**				
Only the 10 annotation clusters that resulted significantly enriched (Enrichment score > 1.3, $p < 0.05$) are reported				
GOTERM_BP_FAT	regulation of ubiquitin-protein ligase activity during mitotic cell cycle	15	7.9×10^{-3}	5.0×10^{-1}
GOTERM_BP_FAT	positive regulation of ligase activity	15	1.0×10^{-2}	5.6×10^{-1}
Annotation Cluster 6	Enrichment Score: 2.11	Count	*p* Value	Benjamini
GOTERM_CC_FAT	Proton-transporting ATP synthase complex, coupling factor F(o)	7	1.8×10^{-3}	3.8×10^{-2}
GOTERM_CC_FAT	Proton-transporting ATP synthase complex	7	1.2×10^{-2}	1.7×10^{-1}
GOTERM_CC_FAT	Proton-transporting two-sector ATPase complex, proton-transporting domain	7	2.3×10^{-2}	2.8×10^{-1}
Annotation Cluster 7	Enrichment Score: 1.99	Count	*p* Value	Benjamini
GOTERM_BP_FAT	Anaphase-promoting complex-dependent proteasomal ubiquitin-dependent protein catabolic process	14	9.1×10^{-3}	5.3×10^{-1}
GOTERM_BP_FAT	negative regulation of ubiquitin-protein ligase activity during mitotic cell cycle	14	9.1×10^{-3}	5.3×10^{-1}
GOTERM_BP_FAT	negative regulation of ligase activity	14	1.2×10^{-2}	5.6×10^{-1}
GOTERM_BP_FAT	negative regulation of ubiquitin-protein ligase activity	14	1.2×10^{-2}	5.6×10^{-1}
Annotation Cluster 8	Enrichment Score: 1.65	Count	*p* Value	Benjamini
SMART	ARF	7	1.2×10^{-2}	8.7×10^{-1}
INTERPRO	ADP-ribosylation factor	7	1.5×10^{-2}	9.9×10^{-1}
INTERPRO	ARF/SAR superfamily	7	6.2×10^{-2}	1.0
Annotation Cluster 9	Enrichment Score: 1.37	Count	*p* Value	Benjamini
UP_SEQ_FEATURE	repeat:WD 2	36	3.9×10^{-2}	1.0
UP_SEQ_FEATURE	repeat:WD 1	36	3.9×10^{-2}	1.0
SP_PIR_KEYWORDS	wd repeat	36	4.0×10^{-2}	5.1×10^{-1}
INTERPRO	WD40 repeat, conserved site	36	5.6×10^{-2}	1.0
Annotation Cluster 10	Enrichment Score: 1.34	Count	*p* Value	Benjamini
GOTERM_CC_FAT	NADH dehydrogenase complex	9	4.3×10^{-2}	4.1×10^{-1}
GOTERM_CC_FAT	respiratory chain complex I	9	4.3×10^{-2}	4.1×10^{-1}
GOTERM_CC_FAT	mitochondrial respiratory chain complex I	9	4.3×10^{-2}	4.1×10^{-1}
GOTERM_MF_FAT	NADH dehydrogenase (quinone) activity	9	5.0×10^{-2}	9.2×10^{-1}
GOTERM_MF_FAT	NADH dehydrogenase (ubiquinone) activity	9	5.0×10^{-2}	9.2×10^{-1}
GOTERM_MF_FAT	NADH dehydrogenase activity	9	5.0×10^{-2}	9.2×10^{-1}

Table S2. *Cont.*

(F)

**GSE43592**				
Only the first 10 annotation clusters are reported (they are all significantly enriched; Enrichment score > 1.3, $p < 0.05$)				
Annotation Cluster 1	Enrichment Score: 5.24	Count	*p* Value	Benjamini
GOTERM_BP_FAT	establishment of RNA localization	30	4.9×10^{-6}	1.2×10^{-3}
GOTERM_BP_FAT	nucleic acid transport	30	4.9×10^{-6}	1.2×10^{-3}
GOTERM_BP_FAT	RNA transport	30	4.9×10^{-6}	1.2×10^{-3}
GOTERM_BP_FAT	RNA localization	30	9.5×10^{-6}	2.1×10^{-3}
Annotation Cluster 2	Enrichment Score: 5.05	Count	*p* Value	Benjamini
INTERPRO	Zinc finger, FYVE-type	15	3.6×10^{-6}	2.8×10^{-3}
SMART	FYVE	15	9.4×10^{-6}	1.7×10^{-3}
INTERPRO	Zinc finger, FYVE-related	15	2.1×10^{-5}	9.9×10^{-3}
Annotation Cluster 3	Enrichment Score: 2.56	Count	*p* Value	Benjamini
UP_SEQ_FEATURE	domain:Rab-GAP TBC	14	1.1×10^{-3}	2.1×10^{-1}
INTERPRO	RabGAP/TBC	14	1.4×10^{-3}	1.9×10^{-1}
SMART	TBC	14	3.0×10^{-3}	2.0×10^{-1}
GOTERM_BP_FAT	regulation of Rab protein signal transduction	14	4.3×10^{-3}	1.7×10^{-1}
GOTERM_BP_FAT	regulation of Rab GTPase activity	14	4.3×10^{-3}	1.7×10^{-1}
GOTERM_MF_FAT	Rab GTPase activator activity	14	4.7×10^{-3}	1.2×10^{-1}
Annotation Cluster 4	Enrichment Score: 2.38	Count	*p* Value	Benjamini
GOTERM_MF_FAT	Protein-lysine *N*-methyltransferase activity	11	4.2×10^{-3}	1.1×10^{-1}
GOTERM_MF_FAT	Histone-lysine *N*-methyltransferase activity	11	4.2×10^{-3}	1.1×10^{-1}
GOTERM_MF_FAT	lysine *N*-methyltransferase activity	11	4.2×10^{-3}	1.1×10^{-1}
Annotation Cluster 5	Enrichment Score: 2.29	Count	*p* Value	Benjamini
UP_SEQ_FEATURE	repeat:WD 2	49	4.4×10^{-3}	4.4×10^{-1}
SP_PIR_KEYWORDS	wd repeat	49	4.5×10^{-3}	7.0×10^{-2}
INTERPRO	WD40 repeat, conserved site	49	8.4×10^{-3}	4.7×10^{-1}
Annotation Cluster 6	Enrichment Score: 2.28	Count	*p* Value	Benjamini
GOTERM_BP_FAT	negative regulation of programmed cell death	63	5.7×10^{-3}	2.1×10^{-1}
GOTERM_BP_FAT	negative regulation of cell death	63	6.0×10^{-3}	2.1×10^{-1}
Annotation Cluster 7	Enrichment Score: 1.95	Count	*p* Value	Benjamini
SMART	PI3K_rbd	5	7.9×10^{-3}	2.3×10^{-1}
INTERPRO	Phosphoinositide 3-kinase, C2	5	1.1×10^{-2}	5.0×10^{-1}
SMART	PI3K_C2	5	1.4×10^{-2}	3.1×10^{-1}
INTERPRO	Phosphatidylinositol Kinase	5	2.6×10^{-2}	7.1×10^{-1}
Annotation Cluster 8	Enrichment Score: 1.85	Count	*p* Value	Benjamini
INTERPRO	FY-rich, C-terminal subgroup	4	6.7×10^{-3}	4.2×10^{-1}
SMART	FYRC	4	8.5×10^{-3}	2.3×10^{-1}
INTERPRO	FY-rich, C-terminal	4	1.5×10^{-2}	5.9×10^{-1}
INTERPRO	FY-rich, N-terminal	4	1.5×10^{-2}	5.9×10^{-1}
INTERPRO	FY-rich, N-terminal subgroup	4	1.5×10^{-2}	5.9×10^{-1}
SMART	FYRN	4	1.9×10^{-2}	3.6×10^{-1}
INTERPRO	MLL Transcription Factor	4	2.8×10^{-2}	7.2×10^{-1}

Table S2. *Cont.*

(F)

**GSE43592**				
Only the first 10 annotation clusters are reported (they are all significantly enriched; Enrichment score > 1.3, $p < 0.05$)				
Annotation Cluster 9	Enrichment Score: 1.82	Count	p Value	Benjamini
INTERPRO	DDE superfamily endonuclease, CENP-B-like	6	1.0×10^{-2}	5.0×10^{-1}
INTERPRO	Centromere protein Cenp-B, helix-turn-helix domain	6	1.5×10^{-2}	5.8×10^{-1}
INTERPRO	Centromere protein Cenp-B, DNA-binding domain 2	6	1.5×10^{-2}	5.8×10^{-1}
SMART	CENPB	6	2.1×10^{-2}	3.7×10^{-1}
Annotation Cluster 10	Enrichment Score: 1.82	Count	p Value	Benjamini
GOTERM_BP_FAT	RNA splicing, via transesterification reactions with bulged adenosine as nucleophile	30	1.5×10^{-2}	3.7×10^{-1}
GOTERM_BP_FAT	nuclear mRNA splicing, via spliceosome	30	1.5×10^{-2}	3.7×10^{-1}
GOTERM_BP_FAT	RNA splicing, via transesterification reactions	30	1.5×10^{-2}	3.7×10^{-1}

(G)

**GSE13732**				
Only the first 10 annotation clusters are reported (they are all significantly enriched; Enrichment score > 1.3, $p < 0.05$)				
Annotation Cluster 1	Enrichment Score: 9.06	Count	p Value	Benjamini
GOTERM_BP_FAT	cellular protein catabolic process	133	3.8×10^{-10}	7.6×10^{-7}
GOTERM_BP_FAT	proteolysis involved in cellular protein catabolic process	132	5.4×10^{-10}	4.3×10^{-7}
GOTERM_BP_FAT	protein catabolic process	133	3.2 × 10−9	2.1×10^{-6}
Annotation Cluster 2	Enrichment Score: 4.81	Count	p Value	Benjamini
UP_SEQ_FEATURE	domain:Helicase C-terminal	31	3.9×10^{-6}	3.7×10^{-3}
INTERPRO	DNA/RNA helicase, C-terminal	31	1.4×10^{-5}	8.6×10^{-3}
SMART	HELICc	31	2.0×10^{-5}	3.6×10^{-3}
INTERPRO	DEAD-like helicase, N-terminal	31	2.1×10^{-5}	1.0×10^{-2}
SMART	DEXDc	31	3.6×10^{-5}	4.3×10^{-3}
Annotation Cluster 3	Enrichment Score: 4.34	Count	p Value	Benjamini
GOTERM_BP_FAT	nuclear mRNA splicing, via spliceosome	39	4.6×10^{-5}	4.6×10^{-3}
GOTERM_BP_FAT	RNA splicing, via transesterification reactions with bulged adenosine as nucleophile	39	4.6×10^{-5}	4.6×10^{-3}
GOTERM_BP_FAT	RNA splicing, via transesterification reactions	39	4.6×10^{-5}	4.6×10^{-3}
Annotation Cluster 4	Enrichment Score: 3.05	Count	p Value	Benjamini
GOTERM_BP_FAT	positive regulation of apoptosis	80	7.4×10^{-4}	4.1×10^{-2}
GOTERM_BP_FAT	positive regulation of programmed cell death	80	9.0×10^{-4}	4.7×10^{-2}
GOTERM_BP_FAT	positive regulation of cell death	80	1.1×10^{-3}	5.3×10^{-2}
Annotation Cluster 5	Enrichment Score: 2.75	Count	p Value	Benjamini
GOTERM_BP_FAT	regulation of apoptosis	134	1.5×10^{-3}	6.4×10^{-2}
GOTERM_BP_FAT	regulation of cell death	135	1.8×10^{-3}	7.3×10^{-2}
GOTERM_BP_FAT	regulation of programmed cell death	134	2.2×10^{-3}	7.9×10^{-2}

Table S2. *Cont.*

(G)

**GSE13732**				
Only the first 10 annotation clusters are reported (they are all significantly enriched; Enrichment score > 1.3, $p < 0.05$)				
Annotation Cluster 6	Enrichment Score: 2.33	Count	*p* Value	Benjamini
GOTERM_BP_FAT	negative regulation of ubiquitin-protein ligase activity during mitotic cell cycle	18	3.1×10^{-3}	1.0×10^{-1}
GOTERM_BP_FAT	Anaphase-promoting complex-dependent proteasomal ubiquitin-dependent protein catabolic process	18	3.1×10^{-3}	1.0×10^{-1}
GOTERM_BP_FAT	negative regulation of ligase activity	18	4.4×10^{-3}	1.3×10^{-1}
GOTERM_BP_FAT	negative regulation of ubiquitin-protein ligase activity	18	4.4×10^{-3}	1.3×10^{-1}
GOTERM_BP_FAT	negative regulation of protein ubiquitination	18	1.2×10^{-2}	2.6×10^{-1}
Annotation Cluster 7	Enrichment Score: 2.26	Count	*p* Value	Benjamini
UP_SEQ_FEATURE	domain:HECT	10	4.2×10^{-3}	5.9×10^{-1}
INTERPRO	HECT	10	5.7×10^{-3}	5.2×10^{-1}
SMART	HECTc	10	6.9×10^{-3}	2.2×10^{-1}
Annotation Cluster 8	Enrichment Score: 1.81	Count	*p* Value	Benjamini
UP_SEQ_FEATURE	domain:SET	13	1.2×10^{-2}	7.9×10^{-1}
INTERPRO	SET	13	1.6×10^{-2}	7.7×10^{-1}
SMART	SET	13	2.0×10^{-2}	4.3×10^{-1}
Annotation Cluster 9	Enrichment Score: 1.79	Count	*p* Value	Benjamini
UP_SEQ_FEATURE	domain:DZF	4	1.4×10^{-2}	8.3×10^{-1}
INTERPRO	DZF	4	1.6×10^{-2}	7.6×10^{-1}
SMART	DZF	4	1.8×10^{-2}	4.1×10^{-1}
Annotation Cluster 10	Enrichment Score: 1.77	Count	*p* Value	Benjamini
GOTERM_BP_FAT	regulation of actin filament polymerization	15	7.6×10^{-3}	1.9×10^{-1}
GOTERM_BP_FAT	regulation of actin polymerization or depolymerization	15	2.2×10^{-2}	3.8×10^{-1}
GOTERM_BP_FAT	regulation of actin filament length	15	2.9×10^{-2}	4.4×10^{-1}

(H)

**Annotation Cluster**	**Recurrence Across Datasets (N/7)**
Nuclear mRNA splicing	5/7
NADH dehydrogenase complex	4/7
RNA localization and transport	2/7
WD40 domain	4/7
ATP synthesis	3/7
Regulation of ubiquitin-protein ligase activity	3/7
Apoptosis	3/7
Phosphoinositide 3-kinase	2/7

Table S3. Summary of the characteristics (**A**) and of the DAVID annotation procedure (**B**) for CAD/MI datasets.

(**A**)

Dataset (Accession Number)	Array Method	Cohort	Tissue	Reference
GSE48060	Affymetrix Human Genome U133 Plus 2.0 Array	31 MI cases 21 controls	Peripheral blood	Suresh *et al.*, 2014 [64]
GSE34198	Illumina human-6 v2.0 expression beadchip	44 MI cases 44 controls	Peripheral blood	* Valenta Z, Mazura I, Kolar M, Grunfeldova H, Feglarova P, Peleska J, Tomeckova M, Kalina J, Zvarova J.
GSE42148	Agilent-028004 SurePrint G3 Human GE 8x60K Microarray	13 CAD cases 11 controls	Whole blood	* Arvind P, Shanker J.
GSE20680	Agilent-014850 Whole Human Genome Microarray 4x44K G4112F	87 CAD cases 52 controls	Whole blood	Elashoff *et al.*, 2011 [65]
GSE20681	Agilent-014850 Whole Human Genome Microarray 4x44K G4112F	99 CAD cases 99 controls	Whole blood	Elashoff *et al.*, 2011 [65]
GSE12288	Affymetrix Human Genome U133A Array	110 CAD cases 112 controls	Peripheral blood	Sinnaeve *et al.*, 2009 [66]
GSE23561	Human 50K Exonic Evidence-Based Oligonucleotide array	6 CAD cases 9 controls	Peripheral blood	Grayson *et al.*, 2011 [67]

(**B**)

Dataset (Accession Number)	N of Identified Cluster	N of Significantly Enriched Clusters **	"Nuclear mRNA Splicing" Category		"NADH Dehydrogenase Complex" Category	
			Cluster Rank	Enrichment Score	Cluster Rank	Enrichment Score
GSE48060	199	12	-	-	-	-
GSE34198	192	9	-	-	-	-
GSE42148	177	7	-	-	-	-
GSE20680	193	6	-	-	-	-
GSE20681	200	7	-	-	-	-
GSE12288	255	15	1	3.04	-	-
GSE23561	198	7	-	-	-	-

* This study has not been published yet (we here report all the contributors indicated in the GEO database); ** Clusters significantly enriched are those showing and enrichment score >1.3 (corresponding to $p < 0.05$); DAVID, Database for Annotation, Visualization and Integrated Discovery (http://david.abcc.ncifcrf.gov/); CAD, Coronary artery disease; MI, Myocardial infarction.

Table S4. The 10 most significantly enriched GO terms for the differentially-expressed genes in MS.

Ranking	ID	Term	*p* Value
1	GO: 0005515	Protein binding	2.6×10^{-54}
2	GO: 0005488	Binding	4.6×10^{-47}
3	GO: 0003824	Catalytic activity	3.2×10^{-25}
4	GO: 1901363	Heterocyclic compound binding	5.1×10^{-24}
5	GO: 0003723	RNA binding	7.7×10^{-23}
6	GO: 0097159	Organic cyclic compound binding	1.2×10^{-22}
7	GO: 0000166	Nucleotide binding	2.1×10^{-21}
8	GO: 1901265	Nucleoside phosphate binding	2.7×10^{-21}
9	GO: 0036094	Small molecule binding	2.7×10^{-21}
10	GO: 00119899	Enzyme binding	1.6×10^{-19}

Table S5. Primers used for genotyping and RT-PCR assays.

Primer	Sequence (5′−3′)	Localization	Application
CELF1−F	GAAGCCAGAAGGAAGGTCCA	Exon 10/11 junction	Semi-quantitative real-time RT-PCR
CELF1−R	TCCCAAAGGGCATAAACATC	Exon 11	
NFAT5−F	(6−FAM)ATCGCCCAAGTCCCTCTACT	Exon 1	Discrimination between NFAT5 Δ2 and full-length isoforms by competitive RT-PCR
NFAT5−R	CACCGCTTGTCTGACTCATT	Exon 3	
rs12599391−F	AGAGGTTGTGGTGAGCCAAG	Intron 2	Genotyping of rs12599391
rs12599391−R	TCACTCTGGGCCAAGTTTTT	Intron 2	
ACTB−F	AGCACAGAGCCTCGCCTTTG	Exon 1	Semi-quantitative real-time RT-PCR (housekeeping gene)
ACTB−R	ACATGCCGGAGCCGTTGT	Exon 2	
HMBS−F	GTTCAGGAGTATTCGGGGAAACC	Exon 8/9 junction	Semi-quantitative real-time RT-PCR (housekeeping gene)
HMBS−R	TTCCTCAGGGTGCAGGATCTG	Exon 9/10 junction	
